# The multifaceted roles of fatty acids and their dysregulation in obese mothers: potential implications for infant development

**DOI:** 10.1186/s12986-025-01009-9

**Published:** 2025-11-13

**Authors:** Boqun Liu, Yuqing Liu, Chuang Zhai, Xuan Wu, Yanmei Wang, Xibi Fang

**Affiliations:** 1 College of food Science and Engineering, Jilin University, Changchun, 130062 China; 2 Department of Clinical Nutrition, Jilin Provincial People’s Hospital, Changchun, 130021 China; 3 Department of Animal Science, Jilin University, Changchun, 130062 China

**Keywords:** Breast milk, Maternal obesity, Fatty acids, Infant health

## Abstract

The increasing global rates of obesity underscore the need to investigate its impact on infant health. Breast milk, crucial for infant nutrition, varies in composition due to maternal obesity during pregnancy. Research reveals that obese or overweight mothers tend to have higher saturated fatty acids (SFAs) levels, like palmitic and myristic acids, while stearic acid levels are lower. Monounsaturated fatty acids (MUFAs), particularly oleic acid in milk, decline in obesity. Polyunsaturated fatty acids (PUFAs), essential for infant brain and nervous system development, show imbalances in obese mothers, with an increased omega-6 (ω-6): omega-3 (ω-3) ratio and reduced levels of key ω-3 fatty acids such as α-linolenic acid (ALA), eicosapentaenoic acid (EPA) and docosahexaenoic acid (DHA). These changes could disrupt normal immune and nervous system development in infants. This review highlights the critical impact of maternal obesity on breast milk quality.

## Introduction

The escalating global epidemic of overweight and obesity—often referred to as “globesity”—is sweeping across many parts of the world. The World Obesity Atlas predicts that by 2035, the obesity rate among adult women worldwide will rise from 18–27% [1, 2]. Maternal obesity affects the health of pregnant women and further influences infant development [3, 4]. Meanwhile, postpartum obesity could lead to changes in the composition of breast milk [5]. Therefore, this article will explore the changes in breast milk fat and their potential impacts on infant health.

**Fig. 1:**
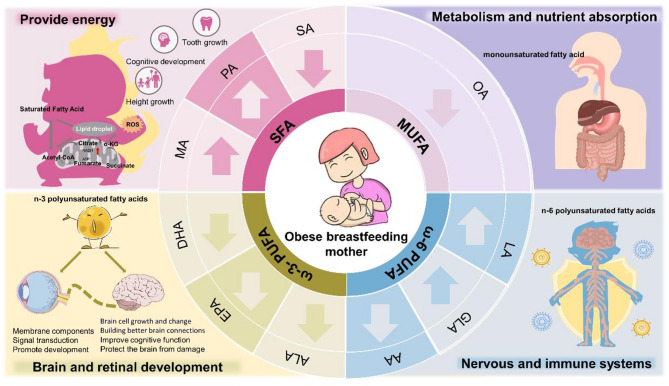
Change trend of fatty acids in breast milk derived from obese or overweight mothers and the main functions of SFA, MUFA, ω−3 and ω−6 PUFA in early infant nutrition. SFA: saturated fatty acid; MUFA: monounsaturated fatty acid; ω−3 PUFA: omega-3 polyunsaturated fatty acid; ω−6 PUFA: omega-6 polyunsaturated fatty acid; MA: myristic acid; PA: palmitic acid; SA: stearic acid; OA: oleic acid; LA: linoleic acid; GLA: γ-linolenic acid; AA: arachidonic acid; ALA: α-linolenic acid

Breast milk is a dynamic, life-giving fluid that provides a full range of nutrients and bioactive factors essential for optimal growth and lifelong health of infants [6–8]. Breast milk fat is the main source of energy for infants, supporting their rapid growth and weight gain [9]. It contains over 200 fatty acids in varying concentrations, with only a few, such as palmitic acid, oleic acid, and linoleic acid, being present in significant amounts [10]. SFAs, such as palmitic acid and stearic acid, primarily provide energy to support the growth and development of newborns [11, 12]. Oleic acid, which is a monounsaturated fatty acid (MUFA), affects cholesterol and energy balance [13–16]. Oleic acid also helps maintain intestinal health and reduce the risk of indigestion [17, 18]. Polyunsaturated fatty acids (PUFAs), especially ω−3 and ω−6 fatty acids, are crucial for the development of the brain, retina, and nervous and immune systems [19, 20], as summarized in Fig. 1.

Overweight or obesity affects fatty acid metabolism, leading to changes in the fatty acid composition of breast milk [21–23]. Breast milk with altered fatty acid levels may lead to excessive weight gain and abnormal fat accumulation during early infancy [24, 25]. This may be associated with an increased risk of obesity, metabolic syndrome, and cardiovascular disease in adulthood [26]. It may also influence cognitive, immune, digestive, and nutritional processes [24]. Therefore, it is crucial to understand how maternal obesity alters the fatty acid composition of breast milk and to uncover the mechanisms behind these changes. This knowledge will help develop effective strategies that improve both maternal and infant health.

This review provides how specific fatty acids—like myristic acid, palmitic acid, stearic acid, oleic acid, linoleic acid, γ-linolenic acid, arachidonic acid, α-linolenic acid, EPA, and DHA—change in the breast milk of overweight or obese mothers, as illustrated in Fig. 1. This work also focuses on how these changes can directly or indirectly affect infant health. The authors emphasize the role of milk fatty acids in supporting infant growth and body functions, aiming to offer guidance for healthy feeding and help prevent childhood obesity and metabolic diseases.

## Saturated fatty acids content changes and infant impacts

Saturated fatty acids (SFAs) dominate the fatty acid composition of breast milk, approximately 44–56% of the total, with the main ones being C8:0, C10:0, C12:0, C14:0, C16:0, C18:0, and C20:0 [27–29]. SFAs, as one of the basic components of lipids, are an essential source of energy for infant growth and development [29]. In the milk of overweight or obese mothers, the composition often shows higher levels of SFAs, particularly myristic acid (C14:0) and palmitic acid (C16:0) [24, 30–32]. Conversely, stearic acid (C18:0) and hydroxy stearate palmitate (PAHSA) are found in lower concentrations in the breast milk of overweight or obese mothers [33, 34]. In this section, we review reported changes in SFAs and their potential implications for infant health.

### Palmitic acid

**Fig. 2:**
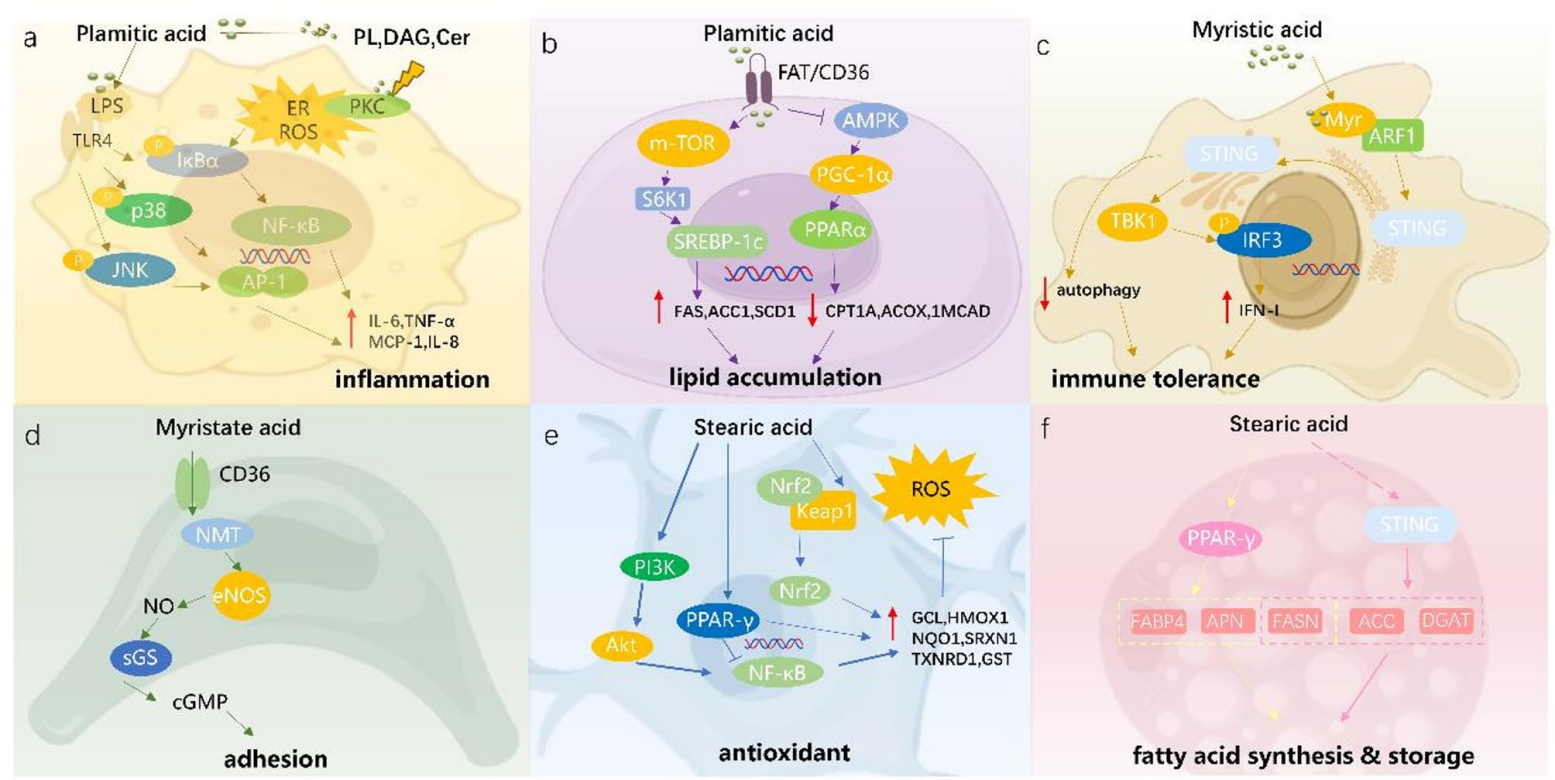
SFAs are involved in multiple signaling pathways regulating cellular metabolism and immune homeostasis. (**a**) Palmitic acid promotes inflammatory responses (**b**) Palmitic acid induces lipid accumulation (**c**) Myristic acid modulates immune tolerance (**d**) Myristic acid regulates vascular function (**e**) Stearic acid confers antioxidant protection (**f**) Stearic acid drives fatty acid synthesis and storage. PL: Phospholipid; DAG: Diacylglycerol; Cer: Ceramide; LPS: Lipopolysaccharide; TLR4: Toll-Like Receptor 4; PKC: Protein Kinase C; JNK: c-Jun N-terminal kinase; p38: p38 MAPK; AP-1: Activator Protein 1; IL-6: Interleukin 6; TNF-α:Tumor Necrosis Factor alpha; MCP-1: Monocyte Chemoattractant Protein 1; ER: Endoplasmic Reticulum stress; ROS: Reactive Oxygen Species production; FAT: Fatty Acid Translocase; S6K1: Ribosomal Protein S6 Kinase 1; PGC-1α:Peroxisome Proliferator-Activated Receptor Gamma Coactivator 1-alpha; FAS: Fatty Acid Synthase; ACC1: Acetyl-CoA Carboxylase 1; SCD1: Stearoyl-CoA Desaturase 1; CPT1A: Carnitine Palmitoyltransferase 1 A; ACOX1: Acyl-CoA Oxidase 1; MCAD: Medium-Chain Acyl-CoA Dehydrogenase; Myr-ARF1: Myristoylated ADP-Ribosylation Factor 1; STING: Stimulator of Interferon Genes; NMT: N-Myristoyltransferase; eNOS: Endothelial Nitric Oxide Synthase; sGS: Soluble Guanylate Cyclase; Keap1: Kelch-like ECH-associated protein 1; Nrf2: Nuclear factor erythroid 2-related factor 2; GCL: Glutamate-Cysteine Ligase; HMOX1: Heme Oxygenase 1; NQO1/SRXN1/TXNRD1/GST: NAD(P)H Quinone Dehydrogenase 1/Sulfiredoxin 1/Thioredoxin Reductase 1/Glutathione S-Transferase; APN: Adiponectin; PTEN: Phosphatase and tensin homolog

Palmitic acid (PA, C16:0) is the primary saturated fatty acid in breast milk, comprising 20–25% of the total fatty acids [35]. High concentrations of PA in breast milk can promote cellular inflammation by inducing inflammatory responses [36]. PA may convert into metabolites such as phospholipids, diacylglycerol, and ceramides, thus activating various Protein Kinase C (PKC) enzymes [36]. These activate PKC, increasing endoplasmic reticulum (ER) stress and reactive oxygen species (ROS) production [37, 38]. And this, in turn, activates Inhibitor of kappa B (IκB) kinase in the NF-κB signaling pathway [39]. PA synergizes with lipopolysaccharide (LPS) to enhance the activity of the Toll-like receptor 4 (TLR4) signaling, leading to the phosphorylation and degradation of IκBα, releasing NF-κB [38]. Once released, NF-κB translocates to the nucleus and activates the transcription of genes involved in inflammatory responses. Furthermore, TLR4 signaling can phosphorylate p38 mitogen-activated protein kinase (p38) and c-Jun N-terminal Kinase (JNK), resulting in the nuclear translocation of Activator Protein 1(AP-1) subunits (e.g., c-fos, c-jun) [40]. In short, PA is associated with pro-inflammatory responses by activating PKC, increasing ER stress and ROS, and enhancing NF-κB and AP-1 signaling through TLR4 pathways (Fig. 2a) [36].

Additionally, PA influences lipid metabolism by regulating fatty acid synthesis and oxidation. It enhances fatty acid synthesis by activating the mTOR/S6K1 and Sterol Regulatory Element-Binding Protein-1c (SREBP-1c) pathways and also inhibits the AMPK/PGC-1α/PPARα signaling pathway to reduce fatty acid oxidation [38, 41]. As a result, it could favor lipid accumulation (Fig. 2b). Moreover, excessive PA in the intestine binds with calcium to form insoluble fatty acid calcium soaps, reducing calcium absorption and potentially leading to infant constipation and impaired bone health [42]. Furthermore, elevated free PA can reduce fat absorption due to calcium soap formation, potentially resulting in lower energy availability [43]. Studies in mice have shown that breast milk high in PA can increase serum IgE in offspring, reduce skin moisture, and induce atopic dermatitis-like symptoms, suggesting that excessive long-chain saturated PA may damage the skin barrier through inflammation. This finding remains to be verified in clinical populations [44]. In summary, high concentrations of PA in breast milk adversely impact inflammatory responses, lipid metabolism, calcium absorption and energy balance in infants [45].

### Myristic acid

Myristic acid (MA, C14:0) is a saturated fatty acid that is typically present at relatively low concentrations in human milk under normal physiological conditions, accounting for approximately 4–8% of total fatty acids [46, 47]. Elevated Levels have been consistently reported in the milk of obese mothers. At appropriate concentrations, MA contributes to infant immune and vascular development; however, excessive exposure may disrupt immune tolerance and vascular homeostasis. Mechanistically, MA enhances the N-myristoylation of ADP ribosylation factor 1 (ARF1), helping to regulate the transport of interferon gene stimulator (STING) from the ER to the Golgi apparatus [48]. STING activates the downstream TANK-binding kinase 1 (TBK1), which in turn phosphorylates interferon regulatory factor 3 (IRF3) [49]. As a result, IRF3 translocates from the cytoplasm to the nucleus and initiates the transcription of type I interferon (IFN-I)-related genes, thereby establishing an antiviral response [50]. Meanwhile, STING-dependent autophagy enhancement and excessive IFN-I can break immune tolerance (Fig. 2c), increasing the risk of autoimmune diseases [50].

In vascular cells, MA can affect the activity of endothelial nitric oxide synthase (eNOS) through CD36, thereby regulating NO signaling [51]. NO stimulates downstream cGMP synthesis and enhances cell adhesion of endothelial cells and vascular smooth muscle cells on type I collagen and other related protein substrates (such as fibronectin) (Fig. 2d) [52]. Cell adhesion affects the behavior of vascular cells and the overall function of blood vessels [51]. In addition, myristic acid also affects the membrane translocation of Fyn kinase and the activation of Src family kinases, interfering with the expression or activity of coagulation-related factors, thereby affecting the coagulation process [52]. MA induced changes in endothelial cell function can affect the normal development and homeostasis of infant blood vessels [53].

In short, MA is present in low levels in breast milk [54]. Synthesised de novo by the mammary gland, MA exerts direct antimicrobial activity against Gram-negative bacteria and selectively promotes *Lactobacillus* colonisation, thereby reinforcing the neonatal gut-immune barrier [44]. In vitro, medium-chain fatty acids, including MA, also attenuate LPS-induced inflammation [46]. Although MA may raise infant cholesterol and pose cardiovascular risk, its physiological levels in breast milk are partly offset by these antimicrobial benefits [55]. However, excessive exposure to MA is not without risk. Elevated levels have been associated with increased infant cholesterol and potential long-term cardiovascular complications, while STING-dependent overactivation may predispose to autoimmune disorders [48, 56]. Therefore, although MA contributes to infant immunity and vascular regulation at physiological concentrations, its excessive intake may disrupt metabolic and immune homeostasis, posing potential health risks comparable to those of other saturated fatty acids [57].

### Stearic acid

Stearic acid (C18:0) is a long-chain saturated fatty acid that makes up approximately 4 to 5.5% of the total fatty acids in breast milk [46, 58, 59]. The level of stearic acid might be reduced due to obesity [60]. Insufficient SA in breast milk is detrimental to the regulation of the nervous system and contributes to abnormal fat metabolism [61]. During early brain development, SFAs in neural lipids are exclusively derived from de novo synthesis. Postnatal intake of SA enables its transport across the blood-brain barrier into neurons via fatty acid-binding proteins (e.g., FABP) [62]. The neuroprotective effects of SA primarily stem from its multimodal regulation of oxidative stress and mitochondrial dynamics [61]. Under oxidative challenge, SA facilitates Nrf2 nuclear translocation by dissociating Keap1/Nrf2 complexes, initiating glutathione synthetase expression [61]. Concurrently, SA activates the PI3K/Akt/NF-κB axis to upregulate antioxidant enzymes (SOD, catalase) and enhances FOXO-mediated redox homeostasis through NAD+-dependent SIRT1 activation (Fig. 2e) [61]. Mitochondrial integrity is preserved via SA-induced stearoylation of TfR1, which suppresses JNK signaling and promotes mitochondrial fusion, thereby sustaining energy metabolism and ROS clearance [63]. Notably, the SA-driven glutathione system not only directly scavenges ROS but also regenerates vitamin C/E, establishing an amplified antioxidant network [61]. These mechanisms reduce oxidative damage and protect neurons by regulating transcription factors, enzymes and organelles, making SA a multifunctional factor for neuroprotection [61].

SA also directly or indirectly activates PPARγ and SREBP-1c [64, 65]. Upon activation, PPARγ initiates adipocyte differentiation and enhances the expression of lipid synthesis enzymes and lipid droplet-associated proteins, including Fatty Acid Synthase (FASN), adiponectin (APN), and Fatty Acid-Binding Protein 4 (FABP4) [66]. By modulating ER stress and intracellular lipid levels, SA indirectly activates SREBP-1c, which subsequently upregulates the expression of genes involved in lipid synthesis and metabolism, such as Acetyl-CoA Carboxylase (ACC), FASN, and Diacylglycerol Acyltransferase (DGAT), thereby enhancing fatty acid synthesis and storage (Fig. 2f) [67]. In summary, SA is essential in early nervous system regulation and in helping newborns build energy reserves. It supports their rapid development and energy needs [65].

SFAs have multiple effects on infants. On the one hand, excessive intake of SFAs in infants is associated with certain diseases. For example, accumulation of unoxidised fatty acids or metabolites increases ER stress and ROS triggers inflammation [68]. Lipid accumulation may contribute to metabolic diseases such as obesity [69]. Calcium malabsorption may contribute to digestive disorders [70]. Although the incidence of cardiovascular disease is low in infancy, chronic intake of excess SFAs increases blood cholesterol levels, especially low-density lipoprotein cholesterol (LDL-C), potentially increasing the risk of cardiovascular disease later in life [71]. On the other hand, persistent deficiencies in the intake of certain SFAs can lead to abnormal physiological functions [72]. The primary role of SFAs is for organismal function, in addition to regulating the immune response, inflammatory response, lipid metabolism, immune function, and neurological development [73–77].

## Potential implications of changes in oleic acid, a representative monounsaturated fatty acid, for infants

Although breast milk contains other monounsaturated fatty acids (MUFAs), oleic acid (OA, C18:1Δ9) is the predominant one, typically accounting for 35%−50% of total fatty acids [78, 79]. Given its crucial roles in energy metabolism, neurodevelopment, and immune regulation, this section focuses on the functions of OA [80–82].

**Fig. 3:**
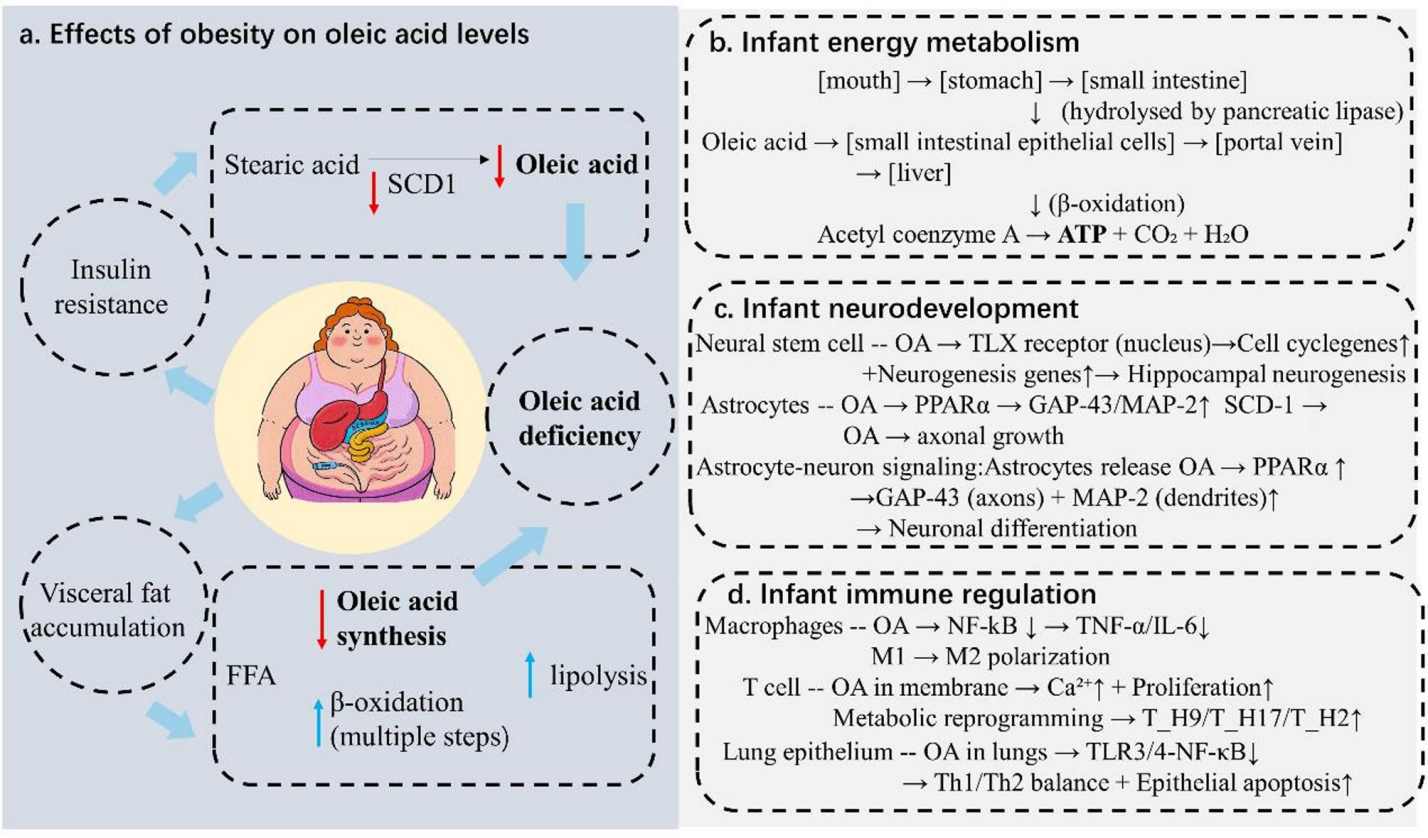
Maternal obesity-mediated imbalance of oleic acid metabolism in breast milk and its multisystemic regulatory mechanism (**a**) Obesity-driven insulin resistance reduces OA bioavailability via stearic acid accumulation (mediated by stearoyl-CoA desaturase 1, SCD1), impaired de novo synthesis from free fatty acids (FFA), and visceral fat sequestration. (**b**) Oleic acid in breast milk is hydrolyzed by pancreatic lipase, absorbed intestinally, and oxidized in the liver to fuel ATP production for infant energy metabolism. (**c**) OA promotes hippocampal neurogenesis by activating neural stem cell proliferation (via thromboxane-like receptor, TXL receptor) and enhances astrocyte-neuron cross-talk, driving axonal and dendritic growth through peroxisome proliferator-activated receptor alpha (PPARα)-dependent upregulation of growth-associated protein 43 (GAP-43, axonal marker) and microtubule-associated protein 2 (MAP-2, dendritic marker). (**d**) OA modulates immune homeostasis by polarizing macrophages (M1/M2 balance), regulating T cell activation via membrane Ca²⁺ signaling, and maintaining pulmonary T helper 1/T helper 2 (Th1/Th2) equilibrium through Toll-like receptor 3/4-nuclear factor kappa B (TLR3/4-NF-κB) pathways. Blue compartments demarcate thematic domains, with red text highlighting molecular targets and black arrows indicating process flow. Visceral fat accumulation and metabolic cross-talk are centrally integrated to emphasize systemic OA dysregulation in obesity

Obesity-induced insulin resistance significantly suppresses the activity of stearoyl-CoA desaturase (SCD1), thereby reducing the efficiency of converting stearic acid (SA) into OA [83]. At the same time, increased visceral fat accumulation promotes lipolysis, resulting in elevated levels of free fatty acids that preferentially undergo β-oxidation [84]. This metabolic shift further limits the biosynthesis of OA (Fig. 3a) [84]. Consequently, the proportion of OA in the breast milk of obese mothers can decline to 15–20%, well below the optimal range [85].

A reduction in OA content may reduce the efficiency of the energy supply to infants and potentially affects their weight gain during early development [86]. OA is primarily located at the sn-1 and sn-3 positions of triglycerides (accounting for approximately 60–70%), where it is readily hydrolyzed by pancreatic lipase and rapidly oxidized to provide energy (Fig. 3b) [87]. When OA levels are insufficient, this efficient energy-release mechanism is disrupted, leading to impaired digestion and absorption of lipids [87]. As a result, infants may experience inadequate energy intake, negatively impacting growth [88].

Beyond its role in energy metabolism, OA plays a critical role in neural development. OA is an endogenous ligand for the orphan nuclear receptor TLX/NR2E1, which governs neural stem and progenitor cell self-renewal and proliferation [89]. Binding of OA converts TLX from a transcriptional repressor to an activator of cell-cycle and neurogenesis genes, thereby enhancing hippocampal neurogenesis in mice [90]. Additionally, OA synthesized in the periventricular zone promotes axonogenesis in the striatum during brain development, with stearoyl-CoA desaturase (SCD1) playing a pivotal role in this process [91]. Furthermore, OA synthesized and released by astrocytes induces neuronal differentiation by upregulating growth-associated protein 43 (GAP-43) and microtubule-associated protein 2 (MAP-2), essential markers for axonal and dendritic growth. This neurotrophic effect of OA is mediated through the activation of peroxisome proliferator-activated receptor-alpha (PPARα), as evidenced by the diminished neuronal differentiation observed when PPARα is silenced (Fig. 3c) [92].

OA also plays an important role in immune regulation. OA exerts significant anti-inflammatory effects by modulating macrophage activity. Specifically, OA decreases the expression of pro-inflammatory cytokines such as TNF-α, IL-6, and IL-1β in LPS-stimulated macrophages, an effect mediated through the inhibition of the NF-κB signaling pathway [93]. Furthermore, OA promotes the polarization of macrophages toward the M2 phenotype, characterized by anti-inflammatory properties. In the context of adaptive immunity, OA influences T cell function by enhancing the proliferation and calcium mobilization of CD4+ T cells upon CD3/CD28 activation [94]. This enhancement is attributed to the incorporation of OA into membrane lipids, which alters membrane fluidity and facilitates signal transduction. Additionally, OA exposure leads to metabolic reprogramming of CD4+ T cells, predisposing them to differentiate into pro-inflammatory subsets, including T_H9, T_H17, and T_H2 cells, upon activation. This effect is linked to the upregulation of genes involved in cholesterol and fatty acid biosynthesis pathways [95]. Moreover, OA has been shown to attenuate asthma pathogenesis by modulating the Th1/Th2 immune balance, suppressing TLR3/4-NF-κB-mediated inflammation, and inducing apoptosis in lung epithelial cells (Fig. 3d) [93].

Taken together, OA in breast milk is a key contributor to infant nutrition and development [29]. Clinical evidence from a Norwegian birth cohort indicates that higher breast-milk oleic acid is linked to an elevated risk of rapid infant growth and fat deposition (OR = 1.10, 95% CI: 1.03–1.19) [96]. Mechanistically, OA remodels the neonatal gut microbiome, while simultaneously supplying structural lipids for neuronal membranes and lowering milk-fat melting points to enhance energy delivery [29]. These integrated actions translate into rapid somatic growth and adipose tissue accrual in early life, implicating OA as a key metabolic programming factor linking maternal lipid supply to infant phenotype and potential later obesity risk [96]. Maintaining physiological OA levels in breast milk is critical for infant metabolic programming and long-term health trajectories [97].

## Polyunsaturated fatty acids content changes and infant impacts

Polyunsaturated fatty acids (PUFAs) are a class of fatty acids containing two or more double bonds [98]. The human body cannot synthesize PUFAs such as linoleic acid (LA) and α-linolenic acid (ALA), which are obtained through diet [98]. Breast milk is the primary source of PUFAs for newborns and infants [99]. Maternal obesity significantly impacts PUFAs levels in breast milk [100]. Specifically, obesity increases linoleic acid (LA) and γ-linolenic acid (GLA). In contrast, α-linolenic acid (ALA), arachidonic acid (AA), EPA, and DHA levels are decreased [24, 46, 101]. This section discusses the changes in each of these PUFAs in detail.

### Omega-3 polyunsaturated fatty acids: DHA and EPA

DHA (C22:6n-3) and EPA (C20:5n-3) are critical bioactive long-chain PUFAs present in breast milk, playing indispensable roles in early neurodevelopment, immune function, and metabolic regulation. A decrease in the levels of DHA and EPA in maternal milk has been associated with suboptimal cognitive development and weakened immune responses in infants [19, 102–105]. One of the central mechanisms involves the phosphorylation and activation of the transcription factor CREB, which leads to the upregulation of Bcl-2, an anti-apoptotic protein essential for neuronal survival [103, 106]. Concurrently, DHA and EPA serve as high-affinity ligands for GPR120, a G protein-coupled receptor. Activation of GPR120 triggers a kinase cascade beginning with phosphorylation of the serine/threonine kinase Raf, which subsequently activates MEK and leads to the phosphorylation of ERK1/2 [107]. Phosphorylated ERK1/2 then translocates into the nucleus to further enhance CREB activity, thus reinforcing neuronal support mechanisms [108].

**Fig. 4:**
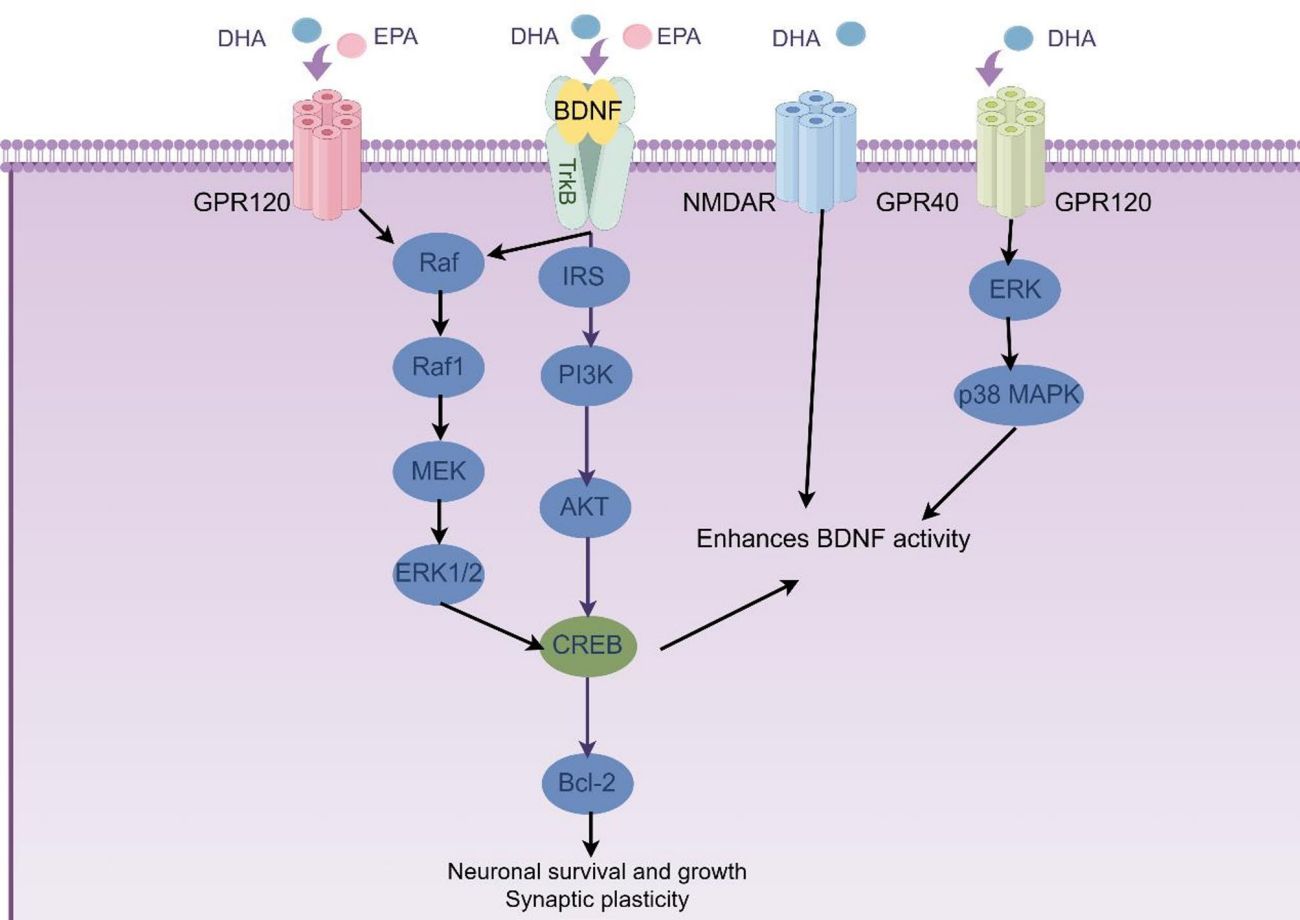
DHA and EPA synergistically regulate neuronal survival and synaptic plasticity through multiple signaling pathways. DHA and EPA bind to GPR120 (G Protein-Coupled Receptor 120), GPR40 (G Protein-Coupled Receptor 40), and NMDAR (N-Methyl-D-Aspartate Receptor), triggering Raf/MEK/ERK, PI3K/AKT, and p38 MAPK cascades. These pathways converge on CREB (cAMP Response Element-Binding Protein) phosphorylation, Bcl-2 (B-cell Lymphoma 2) expression, and BDNF (Brain-Derived Neurotrophic Factor)/TrkB (Tropomyosin Receptor Kinase B) signaling – collectively enhancing neuronal survival and synaptic plasticity

Beyond the CREB axis, DHA contributes to the upregulation of Brain-Derived Neurotrophic Factor (BDNF), a critical neurotrophin for brain development [109]. This regulation may occur indirectly via NMDAR activation, which enhances calcium-dependent signaling pathways, and through interactions with GPR40 and GPR120, which modulate neuroinflammatory and metabolic pathways. BDNF, upon binding to the TrkB receptor, activates downstream MAPK/ERK and PI3K/AKT/CREB pathways, facilitating neuronal survival and synaptic integrity [103, 108–110]. Additionally, BDNF can enhance its own transcription via a CREB-mediated positive feedback loop, further supporting neuroplasticity and cognitive functions (Fig. 4) [103, 111]. Insufficient DHA and EPA intake may disrupt these signaling networks, potentially contributing to suboptimal neural development and immune regulation [105, 112].

Although DHA and EPA share overlapping molecular pathways, they exhibit distinct tissue distribution and functional specializations. DHA is predominantly incorporated into the phospholipids of neural and retinal cell membranes, where it is important for visual and cognitive development [113]. As a structural component of retinal photoreceptor membranes, inadequate DHA levels have been linked to diminished visual acuity in infants [114]. In contrast, EPA is more involved in anti-inflammatory and metabolic regulation [115]. It serves as a precursor for specialized pro-resolving mediators such as resolvins and protectins, which help maintain immune homeostasis [115]. Additionally, EPA can activate AMP-activated protein kinase (AMPK), thereby stimulating PGC-1α-mediated mitochondrial biogenesis, enhancing fatty acid oxidation, and reducing lipid accumulation and insulin resistance [116–118]. A deficiency in EPA may therefore contribute to an increased risk of early-life obesity and metabolic dysregulation [119].

In summary, DHA and EPA work synergistically in breast milk to support infant development across multiple physiological domains [120]. Every 1% increase in cord blood or breast milk DHA has been correlated with modest improvement in optimal neurological scores in newborns [121]. In some clinical trials, mothers supplementing with 600 mg DHA and 140 mg EPA daily have been shown to achieve better scores on the Wechsler Vocabulary, Block Design, and Symbol Search subtests compared to controls at 3–4 Years of age. Their visual evoked potential latency is shortened by approximately 7 ms, which may suggest accelerated maturation of early visual pathways [121]. Anti-inflammatory mechanisms of EPA also indirectly support neurodevelopment: for example, daily EPA intake of 1.6 g EPA or 1.1 g DHA during pregnancy reduces the risk of food allergies and IgE-related eczema in infants by over 30% over the first year [121]. By suppressing pro-inflammatory cytokines, EPA may help support blood-brain barrier integrity, creating a low-inflammatory microenvironment for neural network maturation. DHA primarily ensures optimal neurodevelopment and visual function, while EPA contributes to anti-inflammatory signaling and metabolic stability. Together, these fatty acids form an important foundation for the long-term health and resilience of the infant.

### Omega-3 polyunsaturated fatty acids: ALA

α-Linolenic acid (ALA, C18:3Δ9,12,15), an essential omega-3 fatty acid, plays a fundamental role in infant neurodevelopment and systemic health [122]. Within the body, ALA serves as a precursor for the biosynthesis of long-chain PUFAs such as DHA, which are important for the structural and functional integrity of the brain and retina [123]. A deficiency of ALA in breast milk may therefore lead to reduced EPA and DHA levels in infants, ultimately impairing neural and visual development.

Beyond its role in fatty acid biosynthesis, ALA exerts systemic effects through multiple molecular pathways, and its deficiency may influence multiple pathological processes across organ systems [124]. First, ALA has been shown to regulate the proliferation of intestinal stem cells (ISCs) via activation of the Wnt/β-catenin signaling pathway [125]. Insufficient ALA impairs villus development in the small intestine, leading to compromised nutrient absorption [125]. Second, ALA modulates intestinal microbial homeostasis through the TLR4/NF-κB signaling axis [76]. A decline in ALA levels disrupts this balance, resulting in an increase in LPS leakage, which triggers systemic inflammation and impairs hepatic metabolic function [76].

Taken together, these findings underscore the essential role of ALA in breast milk for supporting optimal neurodevelopment and intestinal health [126]. Ensuring sufficient ALA intake during lactation is therefore critical for promoting the long-term health and cognitive development of the infant.

### Omega-6 polyunsaturated fatty acid: arachidonic acid

Arachidonic acid (AA, C20:4Δ5,8,11,14) is a polyunsaturated fatty acid liberated from membrane phospholipids through the hydrolytic action of phospholipase A2 (PLA2) [127, 128]. As a critical precursor in lipid mediator biosynthesis, AA serves as the substrate for cyclooxygenase (COX) and lipoxygenase (LOX) enzymes, which generate a range of pro-inflammatory compounds, including prostaglandins (PGs), thromboxanes (TXs), and leukotrienes (LTs) [129]. These mediators play essential roles in initiating and regulating immune responses [130]. Therefore, depletion of AA can directly impair their synthesis, leading to dysregulation of immune cell functions—particularly in macrophages and T lymphocytes—and compromising the precision and resolution of inflammatory responses [131, 132].

Interestingly, AA also contributes to the resolution of inflammation through the generation of specialized pro-resolving mediators (SPMs), such as lipoxins, and through the activation of peroxisome proliferator-activated receptor gamma (PPARγ)-dependent signaling pathways. These mechanisms may contribute to the regulation of immunometabolic homeostasis. Experimental studies have demonstrated that AA promotes macrophage polarization toward the anti-inflammatory M2 phenotype via PPARγ activation, thereby facilitating tissue repair while limiting excessive inflammatory responses [133].

In addition to immune modulation, AA also influences cellular proliferation [129, 134]. It has been shown to regulate macrophage cell cycle progression by inducing S-phase arrest through c-Jun N-terminal kinase (JNK) pathway activation, thereby fine-tuning immune cell turnover and proliferation dynamics [135]. Moreover, AA exerts regulatory effects on metabolic processes by inhibiting adipocyte differentiation. This is mediated through suppression of the ERK1/2 signaling pathway and downregulation of COX-1 and COX-2 expression, mechanisms that contribute to the prevention of obesity-associated chronic inflammation [136].

Collectively, these findings underscore the multifaceted regulatory role of AA in maintaining immune and metabolic homeostasis [137, 138]. Through its dual functions in both promoting and resolving inflammation, as well as modulating cell proliferation and adipogenesis, AA plays an indispensable role in coordinating immune responses and preserving systemic physiological balance.

### Omega-6 polyunsaturated fatty acid: linoleic acid

Linoleic acid (LA, C18:2Δ9,12) exists in human breast milk alongside its natural isomer, conjugated linoleic acid (CLA) [139]. LA specifically inhibits key protein tyrosine phosphatases (PTP1B, TC-PTP, and SHP2), attenuating their negative regulation of insulin receptor β-subunit phosphorylation, thereby potentiating insulin receptor signaling [140]. Mechanistically, LA activates both AMP-activated protein kinase (AMPK) and protein kinase B (Akt) signaling cascades, augmenting cellular glucose uptake capacity and improving insulin sensitivity [141, 142]. Notably, elevated LA levels in breast milk (> 2.5% of total fatty acids) Lead to excessive 9-hydroxyoctadecadienoic acid (9-HODE) production, which activates c-Jun N-terminal kinase (JNK) signaling, thereby promoting pro-inflammatory cytokine secretion and disrupting adipocyte metabolic homeostasis in infants [143].

CLA demonstrates potent regulatory effects on adipocyte differentiation and adipose tissue remodeling, suggesting a potential role in obesity pathogenesis [144]. The t10c12-CLA isomer demonstrates dual regulatory capacity by suppressing de novo lipogenesis through the downregulation of fatty acid synthase (FASN) and stearoyl-CoA desaturase (SCD1) while concurrently activating AMPK-mediated energy sensing pathways to inhibit ectopic lipid deposition [145]. Furthermore, CLA enhances adipocyte fatty acid-binding protein (A-FABP) expression via PPARα agonism, facilitating intramuscular triglyceride storage while maintaining systemic lipid homeostasis [146]. Transcriptomic analysis reveals that CLA treatment induces differential expression of 14 Wnt-associated miRNAs (e.g., miR-27b-3p, miR-130a-3p), establishing an epigenetic regulatory network that modulates adipogenic differentiation through β-catenin signaling modulation [147]. In summary, LA is a key regulatory molecule in metabolic-inflammatory homeostasis, and its function is highly dependent on the concentration and metabolic microenvironment.

### Omega-6 polyunsaturated fatty acid: GLA

γ-Linolenic acid (GLA, C18:3Δ6,9,12) is a bioactive omega-6 fatty acid known for its neuroprotective, anti-inflammatory, and metabolic regulatory properties [148–150]. Emerging evidence suggests that GLA can attenuate neuroinflammation induced by amyloid-β (Aβ) peptide through modulation of the NF-κB and MAPK signaling pathways [150]. In addition, GLA exerts anti-inflammatory and metabolic benefits via activation of the TGF-β and peroxisome proliferator-activated receptor gamma (PPARγ) pathways, highlighting its potential to influence both immune and metabolic homeostasis [151, 152]. Furthermore, GLA has been shown to regulate the balance between autophagy and apoptosis by activating the LKB1-AMPK-mTOR signaling cascade, thereby supporting cellular resilience under stress conditions [153].

**Fig. 5:**
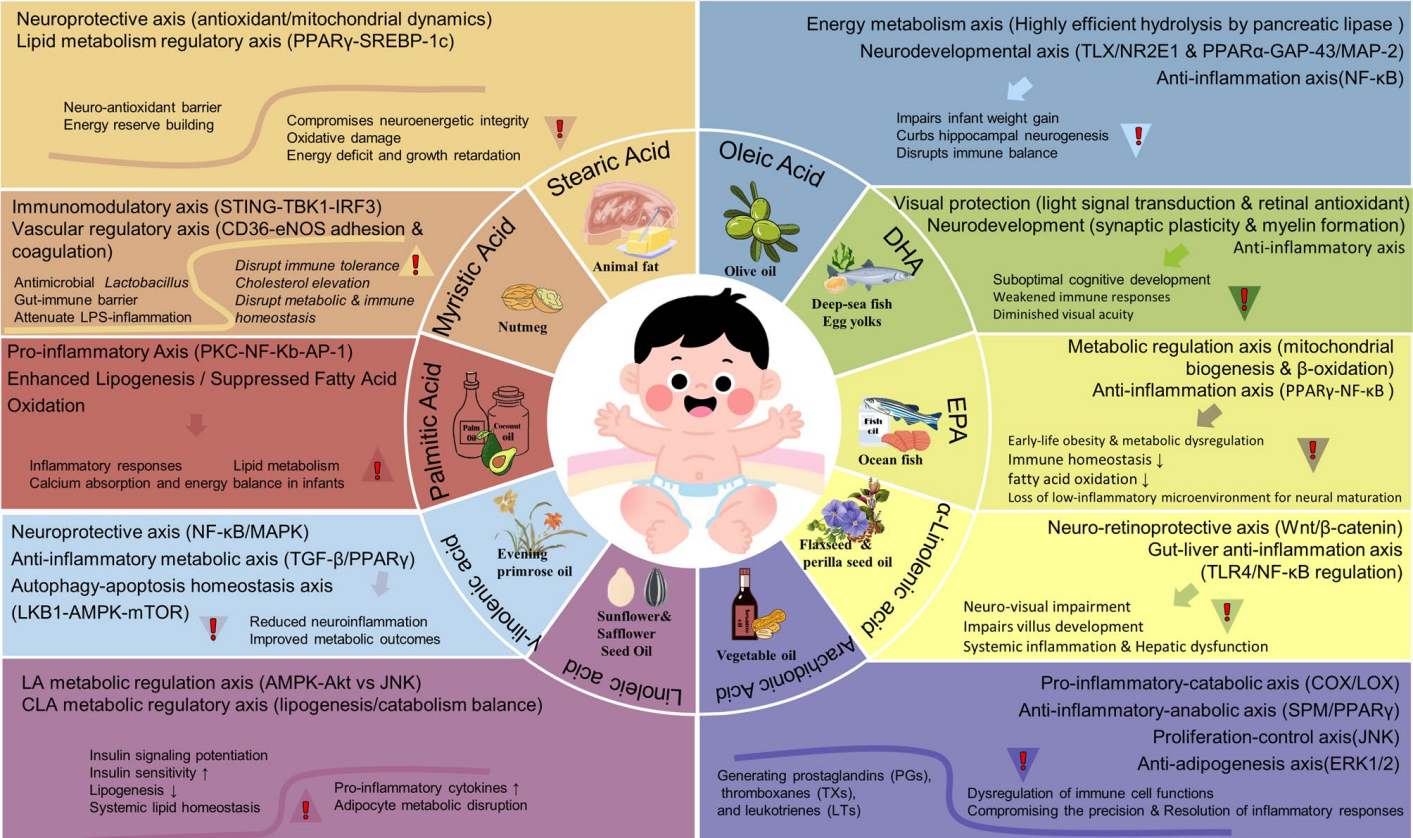
Axis of Molecular Mechanisms Involving Important Fatty Acids in Breast Milk and Outcomes for Infants. Note: The exclamation point refers to the negative effect of an abnormal increase (triangle up) or decrease (triangle down) in this fatty acid, arrows without triangles indicate outcomes observed at normal physiological levels

These multifaceted functions highlight the potential role of GLA in immune regulation, neuroprotection, and the promotion of metabolic health. Elevated GLA levels have been associated with reduced neuroinflammation and improved metabolic outcomes in experimental models [154, 155]. However, clinical evidence on the effects of breast milk GLA concentrations on infant development is still lacking, and further studies are needed to elucidate its early nutritional significance and long-term health effects [150, 152] (Fig. 5).

## Conclusion and future

Obesity or overweight status in pregnant women has emerged as an important factor t of early-life nutritional programming, indirectly influencing infant growth, immune development, and neurocognitive outcomes by altering the fatty acid composition of breast milk. To synthesize the multifaceted molecular mechanisms and infant health implications discussed throughout this review, Figure 5 provides acomprehensive axis integrating these critical relationships. In the context of rising global obesity rates among women of reproductive age, maternal nutritional status during pregnancy and lactation has become an urgent public health concern. The long-term health of infants is increasingly recognized as being shaped not only by genetic and environmental factors but also by the bioactive components of breast milk, particularly its fatty acid profile. This review offers a comprehensive synthesis of current evidence on how specific fatty acids in breast milk mediate their effects through molecular, metabolic, and epigenetic pathways. In doing so, it may provide a foundation for the development of precision nutritional strategies aimed at optimizing maternal health, improving the quality of breast milk, and guiding dietary interventions—including supplementation and complementary feeding—for infants who are not exclusively breastfed.

While existing studies have made significant strides in elucidating the impact of maternal obesity on the concentration of PUFAs, much remains to be explored regarding the broader lipidomic landscape of breast milk. Future research must move beyond the current focus on ω−3 and ω−6 fatty acids to encompass the functional roles of saturated and monounsaturated fatty acids, as well as their interactions with other macronutrients and micronutrients. Understanding how these compositional shifts affect infant metabolic programming, immune resilience, and neurodevelopment may be important for designing effective, evidence-based interventions.

### Knowledge gaps and unresolved questions

Despite notable progress in characterizing the fatty acid alterations associated with maternal obesity, several critical gaps in knowledge persist. One major area of uncertainty is how maternal obesity influences the concentration and bioavailability of other macronutrients in breast milk—particularly amino acids and carbohydrates—which are also essential for infant development. In addition, the long-term consequences of shifts in minor lipid species, such as ether lipids, sphingolipids, and medium-chain fatty acids, remain poorly understood. Their potential contributions to gut maturation, immune training, and microbiota shaping warrant more rigorous investigation.

Although numerous studies have demonstrated the physiological functions of fatty acids in infant development, direct evidence linking maternal obesity-induced alterations in milk fatty acid composition to specific infant developmental outcomes remains limited. This is due to the complexity of separating the effects of maternal metabolic status, dietary intake, and genetic background from those of milk components alone. Furthermore, ethical and practical challenges in human cohort studies make it difficult to establish clear causal relationships. Thus, while the biological plausibility is supported by in *vitro* and animal studies, further well-designed clinical investigations are needed to directly assess how changes in milk fatty acids due to maternal obesity affect infant growth, immune development, or metabolic programming.

Another unresolved question concerns the effectiveness and timing of nutritional interventions designed to correct fatty acid imbalances in the breast milk of obese mothers. Can targeted supplementation during pregnancy or lactation recalibrate the fatty acid profile to one more conducive to favorable infant outcomes? What role might functional foods, dietary patterns, or even microbiota-directed interventions play in this process? Addressing these questions is essential for developing comprehensive, mother-infant-centered nutritional strategies.

Moreover, the current body of literature tends to emphasize the biochemical and physiological dimensions of breast milk composition, often overlooking sociocultural, behavioral, and environmental factors that co-determine maternal nutrition and breastfeeding practices. A more integrated approach is needed—one that situates biochemical research within broader systems of maternal health, dietary accessibility, and healthcare equity.

### Limitations and strategic challenges for future research

While this review synthesizes available evidence, several limitations should be acknowledged. First, many of the included studies are observational, limiting causal inference. Second, much of the mechanistic evidence is derived from animal or in vitro models, which may not fully translate to human physiology. Third, most existing cohorts come from specific geographic or socioeconomic contexts, which restricts generalizability. Finally, by focusing primarily on fatty acids, this review does not fully address the influence of other bioactive milk components, such as proteins, oligosaccharides, or micronutrients, which may interact with fatty acids to shape infant development. These limitations highlight the need for cautious interpretation of current findings and underscore the importance of future large-scale, multi-ethnic, and mechanistically informed studies.

Looking ahead, achieving a holistic understanding of how maternal obesity influences breast milk composition and infant health will require interdisciplinary collaboration across nutrition science, molecular biology, genomics, epigenetics, behavioral science, and sociology. Another major challenge lies in harnessing and interpreting large-scale omics and clinical datasets. As high-throughput sequencing, metabolomics, and lipidomics continue to expand, robust bioinformatics platforms capable of integrating multi-source data will be essential. Building global data-sharing networks that include diverse geographic, ethnic, and socioeconomic populations will be critical for identifying both universal mechanisms and population-specific variations.

Finally, as research advances toward precision nutrition and personalized dietary guidance, the ethical use of personal health and genetic data must remain a priority. Regulatory frameworks will need to evolve alongside technological progress to safeguard data privacy, ensure equity, and promote responsible innovation.

In summary, the relationship between maternal obesity and breast milk composition sits at the nexus of developmental biology, public health, and nutritional science. As we look forward, interdisciplinary, ethically grounded, and globally informed research efforts may be essential for translating mechanistic insights into real-world interventions—ultimately advancing maternal and infant health across diverse populations.

## Data Availability

No datasets were generated or analysed during the current study.
